# Antigenic Patterns and Evolution of the Human Influenza A (H1N1) Virus

**DOI:** 10.1038/srep14171

**Published:** 2015-09-28

**Authors:** Mi Liu, Xiang Zhao, Sha Hua, Xiangjun Du, Yousong Peng, Xiyan Li, Yu Lan, Dayan Wang, Aiping Wu, Yuelong Shu, Taijiao Jiang

**Affiliations:** 1 Center for Systems Medicine, Institute of Basic Medical Sciences, Chinese Academy of Medical Sciences & Peking Union Medical College, Beijing 100005; Suzhou Institute of Systems Medicine, Suzhou, Jiangsu 215123, China; 2 Key Laboratory of Protein and Peptide Pharmaceuticals, Institute of Biophysics, Chinese Academy of Sciences, Beijing 100101, China; 3 National Institute for Viral Disease Control and Prevention, China CDC, Beijing 102206, China; 4 University of the Chinese Academy of Sciences, Beijing 100049, China; 5 Department of Ecology and Evolution, University of Chicago, Chicago, IL 60637; 6 College of Information Science and Engineering, Hunan University, Changsha 410082, China

## Abstract

The influenza A (H1N1) virus causes seasonal epidemics that result in severe illnesses and deaths almost every year. A deep understanding of the antigenic patterns and evolution of human influenza A (H1N1) virus is extremely important for its effective surveillance and prevention. Through development of antigenicity inference method for human influenza A (H1N1), named PREDAC-H1, we systematically mapped the antigenic patterns and evolution of the human influenza A (H1N1) virus. Eight dominant antigenic clusters have been inferred for seasonal H1N1 viruses since 1977, which demonstrated sequential replacements over time with a similar pattern in Asia, Europe and North America. Among them, six clusters emerged first in Asia. As for China, three of the eight antigenic clusters were detected in South China earlier than in North China, indicating the leading role of South China in H1N1 transmission. The comprehensive view of the antigenic evolution of human influenza A (H1N1) virus can help formulate better strategy for its prevention and control.

Seasonal influenza is a long-term threat to human health that causes significant morbidity and mortality every year. Studying the antigenic evolution and seasonal antigenic patterns of human influenza is crucial to formulating effective vaccine strategies.

History of human influenza A (H1N1) can date back to the “Spanish flu” in 1918, which infected almost 500 million^1^,^2^ people around the world and killed about 50 million of them^3^. After being in circulation for nearly 40 years, human influenza A (H1N1) virus disappeared in 1957 after the emergence of human influenza A (H2N2) virus^4^. In 1977, the virus reappeared and has been co-circulating with human influenza A (H3N2) virus ever since^5^. In 2009, a triple reassortant strain of the human influenza A (H1N1) subtype caused severe outbreaks around the world and gradually replaced old lineages^6^.

The long-term epidemic of human influenza A (H1N1) virus is benefited from its fast genetic mutation. To escape the host immune protection, the viral surface antigens underwent frequent mutations and thus antigenic changes. Therefore characterization of the genetic and antigenic evolution of human influenza A (H1N1) is very important for its prevention and control. Masoodi *et al.*^7^, Bragstad *et al.*^8^ and McDonald *et al.*^9^ explored the antigenic and genetic evolution of 2009 pandemic H1N1 virus and some periodical seasonal H1N1 viruses. By using a method named BMDS which combines antigenic maps with genetic information, Bedford *et al.* also assessed the antigenic cartography of H1N1^10^. But its antigenic data were limited, particularly for the viruses in the early years. More recently, by mainly focusing on phylogeny analysis coupled with epidemiological modeling, Trevor *et al.* mapped the global circulation patterns of seasonal H1N1 viruses^11^. Despite the previous efforts, a global view of the antigenic patterns of H1N1 remains unclear.

Here we systematically investigate the antigenic patterns and evolution of the human influenza A (H1N1) virus from 1918 through 2014. We first develop the sequence-based antigenic inference method named PREDAC-H1 based on the PREDAC that we previously developed for modeling the antigenic clusters of human H3N2 viruses. Then we apply PREDAC-H1 to infer the antigenic clusters from a large-scale sequence data covering the whole epidemic history of H1N1. By tracking and comparing the antigenic clusters across different regions, we provide a comprehensive view of the antigenic evolution of the human influenza A (H1N1) virus.

## Results

### Modeling the Antigenic Patterns of the Human Influenza A (H1N1) Virus with PREDAC-H1

**Figure 1:**
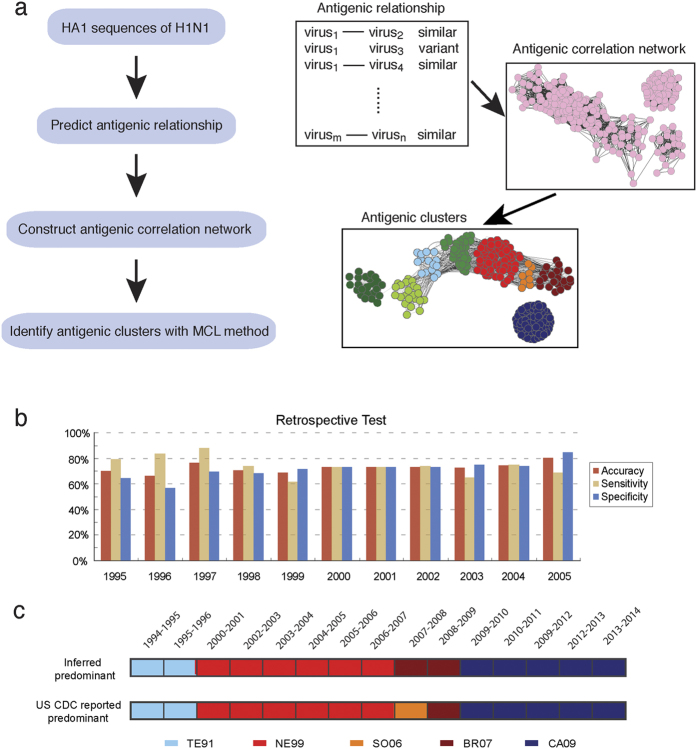
Methodology and validation of the PREDAC-H1 method. (**a**) Workflow of the PREDAC-H1 method, antigenic correlation network was illustrated using Cytoscape software^31^. (**b**) Retrospective testing to infer the antigenic relationships between influenza A (H1N1) viruses. (**c**) Comparison of inferred antigenic clusters and predominant clusters in the US from the 1994–1995 to 2013–2014 epidemic seasons as reported by US CDC. Five dominant clusters are colored and labeled.

In order to model the antigenic patterns of the human influenza A (H1N1) virus, we developed the PREDAC-H1 method. This method was derived from our previous PREDAC method for H3N2^12^, and the workflow is shown in Fig. 1a. There are three key steps in the PREDAC-H1 method. First, the antigenic relationships between pairs of viruses were postulated as being either antigenic variant or similar with a Naïve Bayes model (see Methods and Materials). Then, antigenic similar viruses were used to construct an antigen correlation network (see Methods and Materials). Finally, based on this network, antigenic clusters were identified using the Markov Cluster Algorithm (MCL) method^13^.

In order to test the performance of the PREDAC-H1 method, we collected hemagglutination inhibition (HI) assay data from the Weekly Epidemiological Record (WER) of the WHO. A total of 161 antigenic relationships between 47 viruses were used for further retrospective testing. The results showed a fairly good performance of the PREDAC-H1 method with the average accuracy, sensitivity and specificity reaching 72.6%, 74.1% and 71.2%, respectively (Fig. 1b).

To test whether PREDAC-H1 was able to capture antigenic patterns of H1N1, we defined the predominant cluster of one season as the major antigenic cluster in the given season and further inferred seasonal predominant clusters for the human influenza A (H1N1) viruses surveyed by US CDC (The Centers for Disease Control and Prevention). By comparing actual predominant clusters reported by US CDC (Fig. 1c)^14^, we found that almost all antigenically different vaccine strains were separated into the different antigenic clusters we inferred. Also, these predominant antigenic clusters were consistent with those reported by US CDC. Of the 15 seasons with sufficient sequence data, we accurately inferred 14 of them (Fig. 1c).

### Antigenic Evolution of the Human Influenza A (H1N1) Virus Since 1918

**Figure 2:**
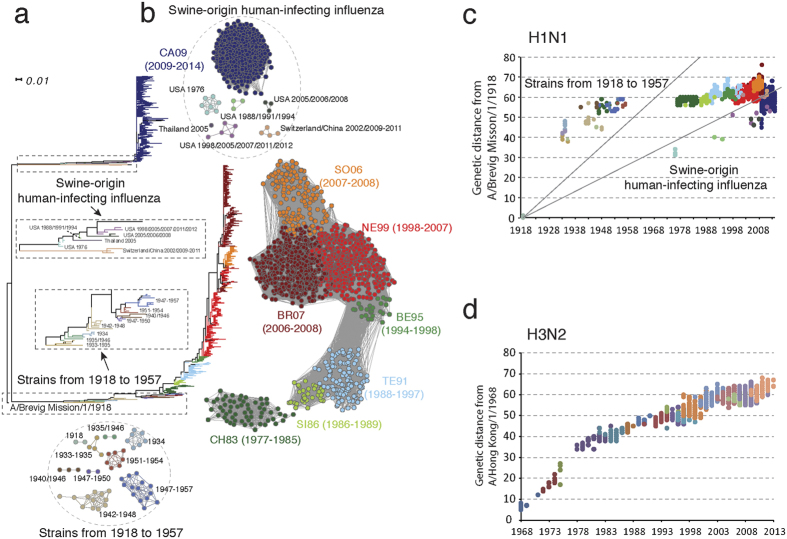
Antigenic and genetic evolution of human influenza A (H1N1) viruses. (**a**) Phylogenetic tree of the HA1 region of the H1N1 HA protein. The tree was rooted using A/Brevig Mission/1/1918 strain as outgroup. (**b**) Inferred antigenic correlation network and antigenic clusters of human influenza A (H1N1) viruses. Clusters are colored and named as the abbreviation of vaccine strains. The circulation periods of each cluster are provided in parenthesis. (**c**) Genetic distance (calculated as amino acid substitutions) on HA1 of human influenza A (H1N1) strains to the A/Brevig Mission/1/1918 (H1N1) virus, each dot represents a virus strain. The strains were colored as antigenic clusters in Fig. 2b. The strains from 1918 to 1957, strains from 1977 to 2008, the swine-origin human-infecting strains were separated by gray lines. (**d**) Genetic distance (calculated as amino acid substitutions) on HA1 of human influenza A (H3N2) strains to the A/Hong Kong/1/1968 (H3N2) virus. Strains were colored as the antigenic clusters they belonged to.

Using the PREDAC-H1 method, we constructed comprehensive antigenic patterns of the seasonal human influenza A (H1N1) virus from 1918 to 2014 (Fig. 2). Two significantly different lineages were observed in the phylogenetic tree (Fig. 2a). One lineage consists of the seasonal human influenza A (H1N1) viruses from 1918 to 2008 and the other was the swine-origin human-infecting influenza A (H1N1) virus, which included the pandemic H1N1 virus in 2009. The first lineage can be divided into 16 antigenic clusters (Fig. 2b). There were nine antigenic clusters from 1918 to 1957 and seven from 1977 to 2008. During the period of 1918–1957, several antigenic clusters co-circulated with each other. For example, there was a main cluster in circulation for 11 years from 1947 to 1957 accompanied by two smaller clusters with duration of 1947–1950 and 1951–1954 respectively. The small cluster of viruses sampled from 1948 to 1950 was antigenically similar to the CH83 cluster, which was consistent with previous studies^15^,^16^.

The seven antigenic clusters between 1977 and 2008 were named according to the vaccine strains they contained. The circulation time of these antigenic clusters ranged from 1 to 10 years (Fig. 2b). The duration of an antigenic cluster was also reflected in the period of vaccine use recommended by WHO. For example, A/New Caledonia/22/1999 was recommended as a vaccine strain from the 2000–2001 season to the 2006–2007 season. CH83 was the main cluster circulating from 1977 to 1985, but this was replaced by SI86 in 1986. SI86 was only dominant for about three years before being replaced by TE91 in 1989. TE91 was the main cluster circulating around the world in the 1990s. In 1994, the BE95 cluster emerged in some regions and circulated for several years. The NE99 cluster emerged in 1998 and was the dominant cluster around the world from 2000 to 2006. Interestingly, the antigenic clusters SO06 and BR07 were two branches that evolved from the NE99 cluster. They co-circulated with each other during 2007 and 2008. After 2009, all previous antigenic clusters died away after the emergence of the CA09 cluster.

We observed that swine-origin human-infecting influenza viruses were sporadic before 2009 and could be divided into several distinct antigenic clusters, which were consistent with previous reports^17^,^18^ (Fig. 2a,b). Notably, the viruses in the same cluster could be collected from different regions and at different time. For example, a small cluster consisted of four viruses from both Switzerland and China that were sampled in 2002, 2009 and 2011, suggestive of sporadic swine to human transmission before 2009. From 2009 to 2014, H1N1pdm formed only one antigenic cluster named as the WHO-recommended vaccine strain CA09, which replaced the anterior seasonal H1N1. Based on the antigenicity inference and phylogenetic analysis, the CA09 was closest to the swine-origin viruses isolated in the US from 1995 to 2012.

As we observed three distinct stages in the antigenic evolution of H1N1, we further analyzed the genetic evolution rates of these stages by comparing to that of human H3N2 (Fig. 2c,d). The genetic evolution of H3N2 was approximately linear while that of human H1N1 was much more complicated and differed significantly among the three stages. From 1918 to 1957, the genetic variation of strains presented approximately linear relationships. As for the genetic evolution of strains from 1977 to 2008, they did not evolved linearly from strains in 1918 since strains in 1977 were antigenically and genetically similar to those around 1950. In terms of swine-origin human-infecting influenza viruses, though the number of strains was quite limited before 2009, we still observed that those strains were approximately linear, with a slope lower than seasonal influenza from 1918 to 1957 and higher than that from 1977 to 2008. We also compared the evolutionary rates in antigen region and non-antigenic region for H1N1 and H3N2 (see Supplementary Fig. S4 online). We found that for both H1N1 and H3N2, the evolutionary rate in antigenic region was much higher than that in non-antigenic region.

### Antigenic Patterns of the Human Influenza A (H1N1) Virus in Different Regions

**Figure 3:**
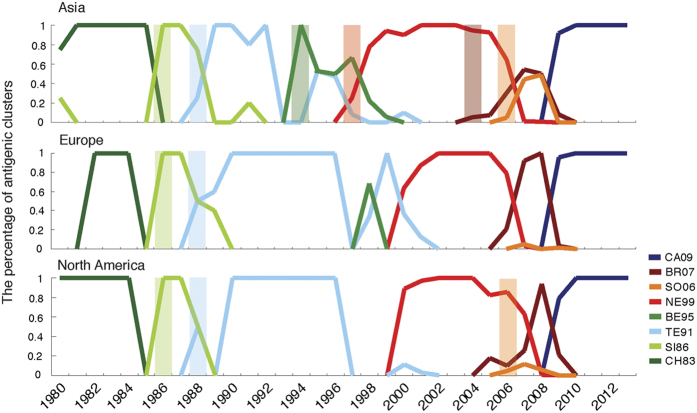
Comparison of antigenic patterns in different regions from 1980 to 2013. Dynamic changes in the percentage of antigenic clusters in Asia, Europe and North America were recorded yearly. The earliest appearance of a new antigenic cluster is marked by a colored bar.

In order to study detailed antigenic patterns in different regions, we mapped antigenic clusters of human influenza A (H1N1) viruses in Asia, Europe and North America. Detailed predominant clusters during each year are shown in Fig. 3. The earliest emergence of each antigenic cluster is marked by a colored bar. Here, we defined the emergence of a new antigenic cluster if the new antigenic cluster emerged with cluster percentage above 5%. Most clusters after 1990 first emerged in Asia. The BE95, NE99 and BR07 antigenic clusters were first detected in Asia and the SO06 cluster was first detected in Asia and North America. The SO06 cluster circulated in conjunction with the BR07 cluster in Asia from 2007 to 2009. The BE95 cluster caused a long-term epidemic in Asia (including China), but was only dominant in Europe for one year (1998) and was not detected in North America during any year, according to sequence data. This showed the low activity of the BE95 cluster in both Europe and North America. The SO06 cluster was not predominant in Europe or North America during any year. The early appearance of most new antigenic variants and the diversity of antigenic clusters in Asia reinforce the origin of influenza variants in this region.

After further analysis of detailed antigenic patterns in Asia, we found that China played an important role in transmission of the human influenza A (H1N1) virus. The CH83 cluster first appeared in China a year before it appeared in other regions of Asia. The SO06 cluster was the dominant antigenic cluster from 2006 to 2008 while the BR07 cluster was dominant in other regions of Asia, Europe and North America. The SO06 cluster also appeared in China earlier than it appeared in other regions of Asia (see Supplementary Fig. S2 online). According to our previous work, most antigenic clusters of the human influenza A (H3N2) virus also first appeared in China, and some were only dominant in this country^12^.

### Circulation of the Human Influenza A (H1N1) Virus in Different Regions of China

**Figure 4:**
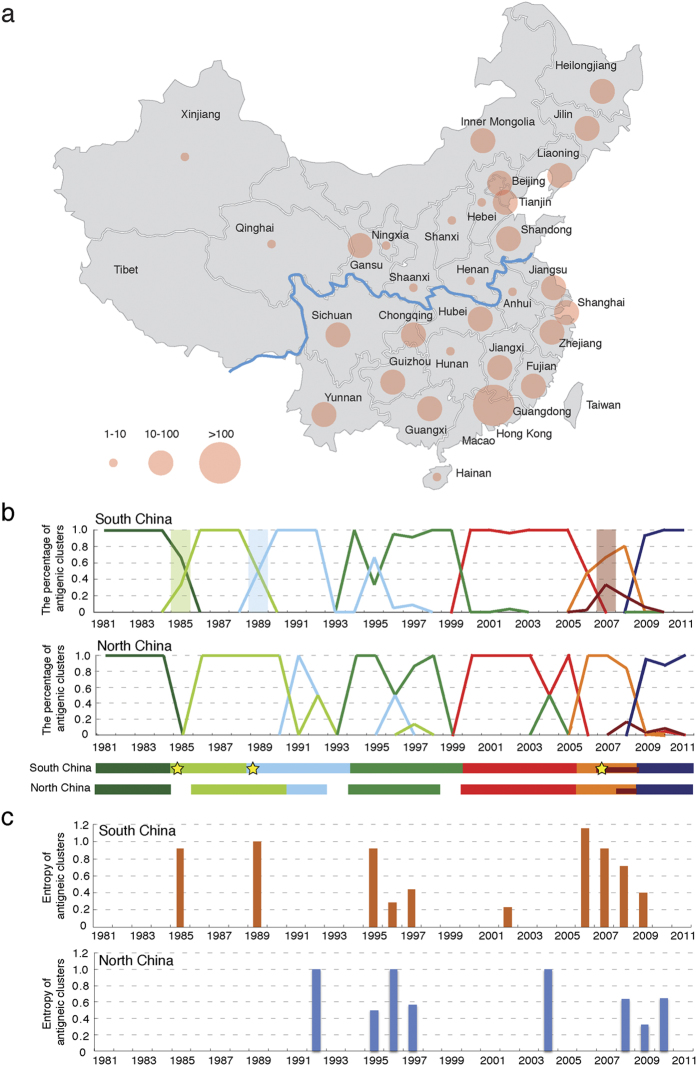
Antigenic patterns in South and North China. (**a**) Geographic distribution of H1N1 HA sequences provided by the China CDC and obtained from a public database from 1977 to 2008. The map was reconstructed using OpenStreetMap (http://www.openstreetmap.org/) with further modification, and is for illustrative purposes only. (**b**) Dynamic changes in the percentage of antigenic clusters in South and North China. The underlying bar represents the circulation period of each antigenic cluster in South and North China and the earliest appearance of a new antigenic cluster is marked with a star. (**c**) Co-circulation of antigenic clusters in South and North China. Co-circulation was measured as the entropy of co-circulating antigenic clusters.

As we have demonstrated, China played a leading role in the circulation of the human influenza A (H1N1) virus. To gain a deep understanding of the antigenic evolution of human H1N1 inside China, the Chinese Center for Disease Control and Prevention (China CDC) has conducted large-scale sequencing of HA segments from representative regions of China during influenza surveillance. By combining these data with those collected from a public database (see Materials and Methods), we further mapped the antigenic evolution of influenza H1N1 in different regions of China (see Supplementary Fig. S1 online). China can be divided into two regions, South and North China with different climates and geographical traits, by the Huai River-Qin Mountains line (Fig. 4a).

We mapped the antigenic evolution of H1N1 in South and North China (Fig. 4b). From 1981 to 2011, there were seven antigenic clusters including CA09 in circulation in both regions of the country. Of these seven antigenic clusters, SO06 and CA09 were detected in the same year in both South and North China, and the exact time of the BE95 and NE99 clusters in North China could not be located due to missing data. While for the other three antigenic clusters (SI86, TE91 and BR07), they emerged earlier in South China than in North China.

It was also observed the circulation patterns were much more complex in South China. The co-circulation of two different antigenic clusters (either with a percentage above 30%) was discovered in both South and North China. We defined the complexity of co-circulation as co-circulation entropy (see Methods and Materials) and plotted the entropy value for each year in South and North China (Fig. 4c). In 1985, 1989 and 2006, there observed co-circulation of both newly emerged antigenic clusters and old antigenic clusters in South China. Although the new antigenic cluster BE95 emerged and became dominant since 1994, the old TE91 cluster re-appeared and reclaimed the predominance in 1995 and 1996 in South China and North China respectively. In 2007, there emerged two new antigenic clusters (namely BR07 and SO06) in co-circulation in South China. In North China, there also observed co-circulation of two antigenic clusters in 1992, 1996 and 2004.

## Discussion

By developing and using the sequence-based antigenicity inference approach PREDAC-H1, we systematically identified the antigenic clusters of human influenza A (H1N1) and analyzed its antigenic evolution. With large scale HA sequencing of H1N1 in China, we further mapped the detailed antigenic patterns in China.

Given that there exists some bias distribution of the sequence data, the antigenic patterns inferred based on sequence data available could not reflect perfectly the actual epidemics. But nevertheless our method provides a preferable way to correlate sequence data with influenza circulation and the expected results correlated well with the US CDC reports. Due to the rapid development of sequencing technology and improved surveillance strategies, sequence samples will become more reliable and the inferred antigenic clusters will reflect the actual epidemics more accurately. Recently, some methods were developed to predict the predominant H3N2 strains in the next season^19^,^20^. Similarly, our sequence-based antigenicity inference method could be further developed into a prediction method with proper modification.

The average replacement cycle of antigenic clusters of H1N1 and H3N2 was 4.6 years and 3.3 years^21^, respectively, which shows that H1N1 experienced much slower antigenic evolution. Asia (including China) is thought to be an important region for the transmission of influenza, and some previous studies^22^,^23^ have demonstrated the leading role Asia plays in the transmission of H3N2. Our results indicated that the antigenic pattern of human influenza A (H1N1) was more complex in Asia and most new antigenic clusters first appeared in this region. Some clusters are predominant in Asia, only causing small epidemics in Europe and North America. The southern region of China may play an important role in the seeding and transmission of influenza due to the earlier emergence of most antigenic clusters. Those findings were consistent with Trevor *et al.*’s work^11^ which showed that most lineages of H1N1 eventually coalesced with viruses from East and Southeast Asia and India with the geographic segregation.

Quite different from H3N2, H1N1 demonstrated extensive co-circulation of different antigenic clusters. Intriguingly, the resurgence of some old clusters was also observed even after a new antigenic cluster had become predominant for a while (Fig. 3). For example, in Asia, SI86 cluster reappeared in 1991 after being replaced by TE91 cluster in 1989. The co-circulation was even more complicated in Asia since H1N1 was much more active here than in other regions.

Our work also highlights the necessity of region-specific H1N1 vaccine recommendations. Our analyses showed that two of the seven H1N1 antigenic clusters during 1977–2008 mainly dominated in Asia. For example, the BE95 cluster dominated in Asia from 1994 to 1997, dominated in Europe in 1998 and never dominated in North America. The similar phenomenon was also discovered in the evolution of H3N2^12^. The JX06 antigenic cluster of H3N2 was dominant in China in the 2006–2007 season, but it didn’t dominate in the United States or Europe. No doubt, characterization of the antigenic patterns of H1N1 in different regions and the further study of the co-circulation patterns of H1N1 and H3N2 are helpful to formulating better surveillance strategies.

## Methods and Materials

### HA Sequence Data

Sequence data was obtained from the Influenza Virus Sequence Database of the NCBI^24^. Chinese data was too limited to be able to obtain a more detailed description of antigenic evolution in China, so we obtained more sequence data from the China CDC and the Global Initiative on Sharing All Influenza Data (GISAID). For sequences with same name, we only selected one of them. All HA1 sequences were aligned with ClustalW^25^. A phylogenetic tree was constructed using PhyML^26^ and displayed using Dendroscope^27^.

### Hemagglutination Inhibition (HI) Data for the Human Influenza A (H1N1) Virus

We collected a dataset of HI measurements from the Weekly Epidemiological Record (Supplementary Table S1 online for detailed information) of the WHO. We then used Archetti-Horsfall distance (dAH)^28^ to define the antigenic relations between viruses, which is defined as follows:


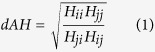


Where H_ij_ refers to the HI titer of strain *i* relative to antisera raised against strain *j*. A pair of viruses were considered antigenic similar if dAH < 4 (as in Liao’s work^29^), otherwise they were considered antigenic different. For pairs with multiple HI test results, we used the median of our dataset. In total, we obtained 70 antigenic variant pairs and 91 antigenic similar pairs.

### Naïve Bayesian Model to Infer the Antigenicity of the Human Influenza A (H1N1) Virus

The first step in modeling the antigenic evolution of the human influenza H1N1 virus was accurately inferring the antigenic relationship between two viruses. We developed a feature-based model of human influenza H3N2^12^ that took into account the structural and physicochemical features that underline antigen-antibody interaction. We adapted it to the human influenza H1N1 virus by making two modifications, using the epitopes of H1N1^30^ and the HI dataset described above as a training dataset. Based on the training dataset, we calculated a threshold cut-off for each feature, and then built a Naïve Bayes classifier to infer antigenic relationships, as we performed in the earlier work^12^. In 5-fold validations, the accuracy rate of the model was 82%. The viruses in the HI dataset were sampled from 1977 to 2007, but the number of virus pairs before 1995 and after 2005 were quite limited so we only conducted retrospective testing for the period from 1995 to 2005. For 1995, we used pairs in which both viruses were collected before 1995 (including 1995) as the training dataset and the remaining pairs as the testing dataset.

### Mapping the Antigenic Clusters of the Human Influenza A (H1N1) Virus

We used the computational PREDAC-H1 method to model antigenic clusters of H1N1 viruses. The antigenic relationship between each pair of viruses in a group of H1N1 viruses was inferred based on their HA sequences. Then, we constructed an antigenic correlation network (ACnet)^12^ by connecting pairs of viruses inferred to be similar in their antigenicity. Groups of viruses with similar antigenicity, denoted as expected antigenic clusters, could then be identified from the ACnet using MCL (see Supplementary Fig. S3 online for detailed selection of inflation parameters).

### Co-circulation Entropy

Co-circulation entropy is defined as:


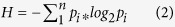


Where p_i_ refers to the percentage of an antigenic cluster in one year. The value of this parameter reflects the degree of co-circulation of antigenic clusters.

## Additional Information

**How to cite this article**: Liu, M. *et al.* Antigenic Patterns and Evolution of the Human Influenza A (H1N1) Virus. *Sci. Rep.* **5**, 14171; doi: 10.1038/srep14171 (2015).

## Supplementary Material

Supplementary Information
