# Supplementary material for: Antigenic Patterns and Evolution of the Human Influenza A (H1N1) Virus
Source: Sci Rep. 2015 Sep 28;5:14171. doi: 10.1038/srep14171 (PMC4585932; doi:10.1038/srep14171)
Supplement: Supplementary Information [file srep14171-s1.pdf]

Supplementary Materials for

Antigenic Patterns and Evolution of the Human Influenza A (H1N1) Virus

Mi Liu, Xiang Zhao, Sha Hua, Xiangjun Du, Yousong Peng, Xiyan Li, Yu Lan, Dayan Wang, Aiping Wu#, Yuelong Shu#, Taijiao Jiang#

#correspondence to:

taijiao@moon.ibp.ac.cn (T.J)

yshu@cnic.org.cn (Y.S)

wuaiping@moon.ibp.ac.cn (A.W)

This PDF file includes:

Supplementary Figures S1 to S4

Supplementary Table S1

Supplementary Note S1

Antigenic strain 1	Antigenic strain 2	Antigenic relationship*
A/USSR/90/1977	A/Bayern/7/1995	1
A/USSR/90/1977	A/Brazil/11/1978	0
A/USSR/90/1977	A/Chile/1/1983	1
A/USSR/90/1977	A/India/6263/1980	1
A/USSR/90/1977	A/New Caledonia/20/1999	1
A/USSR/90/1977	A/Singapore/6/1986	1
A/USSR/90/1977	A/Taiwan/1/1986	1
A/USSR/90/1977	A/Texas/36/1991	1
A/Brazil/11/1978	A/Bayern/7/1995	1
A/Brazil/11/1978	A/India/6263/1980	1
A/Brazil/11/1978	A/New Caledonia/20/1999	1
A/Brazil/11/1978	A/Singapore/6/1986	1
A/Brazil/11/1978	A/Taiwan/1/1986	1
A/Brazil/11/1978	A/Texas/36/1991	1
A/India/6263/1980	A/Chile/1/1983	1
A/Chile/1/1983	A/Bayern/7/1995	1
A/Chile/1/1983	A/Brazil/11/1978	0
A/Chile/1/1983	A/New Caledonia/20/1999	1
A/Chile/1/1983	A/Singapore/6/1986	1
A/Chile/1/1983	A/Taiwan/1/1986	1
A/Chile/1/1983	A/Texas/36/1991	1
A/Singapore/6/1986	A/Bayern/7/1995	0
A/Singapore/6/1986	A/New Caledonia/20/1999	1
A/Singapore/6/1986	A/Sichuan/4/1988	0
A/Singapore/6/1986	A/Taiwan/1/1986	0
A/Singapore/6/1986	A/Texas/36/1991	0
A/Taiwan/1/1986	A/Bayern/7/1995	0
A/Taiwan/1/1986	A/Beijing/262/1995	1
A/Taiwan/1/1986	A/New Caledonia/20/1999	1
A/Taiwan/1/1986	A/Sichuan/4/1988	0
A/Taiwan/1/1986	A/Singapore/15/1996	1
A/Taiwan/1/1986	A/Texas/36/1991	0
A/Taiwan/1/1986	A/Wuhan/371/1995	1
A/Sichuan/4/1988	A/Texas/36/1991	0
A/Texas/36/1991	A/Auckland/176/1999	1
A/Texas/36/1991	A/Bayern/7/1995	0
A/Texas/36/1991	A/Beijing/262/1995	1
A/Texas/36/1991	A/Johannesburg/82/1996	0
A/Texas/36/1991	A/New Caledonia/20/1999	1
A/Texas/36/1991	A/Singapore/15/1996	1

A/Texas/36/1991	A/Wuhan/371/1995	1
A/Wuhan/371/1995	A/Bayern/7/1995	1
A/Wuhan/371/1995	A/Beijing/262/1995	0
A/Wuhan/371/1995	A/Johannesburg/159/1997	0
A/Wuhan/371/1995	A/New Caledonia/20/1999	1
A/Wuhan/371/1995	A/Singapore/15/1996	0
A/Bayern/7/1995	A/Beijing/262/1995	1
A/Bayern/7/1995	A/Johannesburg/159/1997	1
A/Bayern/7/1995	A/Johannesburg/82/1996	0
A/Bayern/7/1995	A/Singapore/15/1996	1
A/Beijing/262/1995	A/Arizona/1/2006	0
A/Beijing/262/1995	A/Auckland/176/1999	0
A/Beijing/262/1995	A/Buenos Aires/344/1999	1
A/Beijing/262/1995	A/Chiba/1042/1999	0
A/Beijing/262/1995	A/Florida/3/2004	0
A/Beijing/262/1995	A/Florida/4/2004	0
A/Beijing/262/1995	A/Hong Kong/2070/1999	0
A/Beijing/262/1995	A/Jiangxi/160/2005	1
A/Beijing/262/1995	A/Johannesburg/159/1997	0
A/Beijing/262/1995	A/Johannesburg/82/1996	1
A/Beijing/262/1995	A/Kentucky/1/2005	0
A/Beijing/262/1995	A/Malaysia/1513/2004	0
A/Beijing/262/1995	A/New Caledonia/20/1999	0
A/Beijing/262/1995	A/New Caledonia/9/2004	0
A/Beijing/262/1995	A/New York/1/2003	0
A/Beijing/262/1995	A/Pennsylvania/1/2006	0
A/Beijing/262/1995	A/Peru/1621/1999	0
A/Beijing/262/1995	A/Singapore/107/2005	1
A/Beijing/262/1995	A/Singapore/14/2004	0
A/Beijing/262/1995	A/Texas/1/2003	1
A/Beijing/262/1995	A/Yamaguchi/12/2002	1
A/Johannesburg/82/1996	A/Auckland/176/1999	1
A/Johannesburg/82/1996	A/Buenos Aires/344/1999	0
A/Johannesburg/82/1996	A/Chiba/1042/1999	1
A/Johannesburg/82/1996	A/Hong Kong/2070/1999	1
A/Johannesburg/82/1996	A/Johannesburg/159/1997	0
A/Johannesburg/82/1996	A/New York/1/2003	1
A/Johannesburg/82/1996	A/Peru/1621/1999	1
A/Johannesburg/82/1996	A/Texas/1/2003	1
A/Johannesburg/82/1996	A/Wuhan/371/1995	1
A/Johannesburg/82/1996	A/Yamaguchi/12/2002	1
A/New Caledonia/20/1999	A/Alabama/22/2006	0
A/New Caledonia/20/1999	A/Alabama/23/2006	0

A/New Caledonia/20/1999	A/Arizona/1/2006	0
A/New Caledonia/20/1999	A/Auckland/176/1999	0
A/New Caledonia/20/1999	A/Bayern/7/1995	1
A/New Caledonia/20/1999	A/Brisbane/59/2007	1
A/New Caledonia/20/1999	A/Buenos Aires/344/1999	1
A/New Caledonia/20/1999	A/Chiba/1042/1999	0
A/New Caledonia/20/1999	A/Florida/13/2007	1
A/New Caledonia/20/1999	A/Florida/19/2007	1
A/New Caledonia/20/1999	A/Florida/3/2004	0
A/New Caledonia/20/1999	A/Florida/4/2004	0
A/New Caledonia/20/1999	A/Fukushima/141/2006	1
A/New Caledonia/20/1999	A/Hong Kong/2070/1999	0
A/New Caledonia/20/1999	A/Hong Kong/2652/2006	1
A/New Caledonia/20/1999	A/Jiangxi/160/2005	1
A/New Caledonia/20/1999	A/Johannesburg/82/1996	1
A/New Caledonia/20/1999	A/Kentucky/1/2005	0
A/New Caledonia/20/1999	A/Kentucky/2/2006	0
A/New Caledonia/20/1999	A/Malaysia/1513/2004	0
A/New Caledonia/20/1999	A/Missouri/12/2006	0
A/New Caledonia/20/1999	A/New Caledonia/9/2004	0
A/New Caledonia/20/1999	A/New York/1/2003	0
A/New Caledonia/20/1999	A/Pennsylvania/1/2006	0
A/New Caledonia/20/1999	A/Pennsylvania/14/2007	1
A/New Caledonia/20/1999	A/Peru/1621/1999	0
A/New Caledonia/20/1999	A/Singapore/107/2005	0
A/New Caledonia/20/1999	A/Singapore/14/2004	0
A/New Caledonia/20/1999	A/Singapore/66/2006	1
A/New Caledonia/20/1999	A/Solomon Islands/3/2006	1
A/New Caledonia/20/1999	A/Texas/1/2003	0
A/New Caledonia/20/1999	A/Texas/25/2006	1
A/New Caledonia/20/1999	A/Virginia/1/2006	0
A/New Caledonia/20/1999	A/Yamaguchi/12/2002	1
A/Jiangxi/160/2005	A/Arizona/1/2006	1
A/Jiangxi/160/2005	A/Pennsylvania/1/2006	0
A/Jiangxi/160/2005	A/Singapore/107/2005	0
A/Kentucky/1/2005	A/Arizona/1/2006	0
A/Kentucky/1/2005	A/Jiangxi/160/2005	1
A/Kentucky/1/2005	A/Pennsylvania/1/2006	0
A/Kentucky/1/2005	A/Singapore/107/2005	0
A/Kentucky/2/2006	A/Alabama/22/2006	0
A/Kentucky/2/2006	A/Alabama/23/2006	0
A/Kentucky/2/2006	A/Hong Kong/2652/2006	1
A/Kentucky/2/2006	A/Missouri/12/2006	0

A/Kentucky/2/2006	A/Singapore/66/2006	0
A/Kentucky/2/2006	A/Solomon Islands/3/2006	0
A/Kentucky/2/2006	A/Texas/25/2006	1
A/Kentucky/2/2006	A/Virginia/1/2006	0
A/Fukushima/141/2006	A/Florida/13/2007	0
A/Fukushima/141/2006	A/Florida/19/2007	0
A/Fukushima/141/2006	A/Pennsylvania/14/2007	0
A/Hong Kong/2652/2006	A/Alabama/22/2006	1
A/Hong Kong/2652/2006	A/Alabama/23/2006	0
A/Hong Kong/2652/2006	A/Missouri/12/2006	1
A/Hong Kong/2652/2006	A/Singapore/66/2006	0
A/Hong Kong/2652/2006	A/Solomon Islands/3/2006	0
A/Hong Kong/2652/2006	A/Texas/25/2006	0
A/Solomon Islands/3/2006	A/Alabama/22/2006	0
A/Solomon Islands/3/2006	A/Alabama/23/2006	0
A/Solomon Islands/3/2006	A/Brisbane/59/2007	0
A/Solomon Islands/3/2006	A/Florida/13/2007	0
A/Solomon Islands/3/2006	A/Florida/19/2007	0
A/Solomon Islands/3/2006	A/Fukushima/141/2006	0
A/Solomon Islands/3/2006	A/Missouri/12/2006	0
A/Solomon Islands/3/2006	A/Pennsylvania/14/2007	0
A/Solomon Islands/3/2006	A/Singapore/66/2006	0
A/Solomon Islands/3/2006	A/Texas/25/2006	0
A/Virginia/1/2006	A/Alabama/22/2006	0
A/Virginia/1/2006	A/Alabama/23/2006	0
A/Virginia/1/2006	A/Hong Kong/2652/2006	1
A/Virginia/1/2006	A/Missouri/12/2006	0
A/Virginia/1/2006	A/Singapore/66/2006	0
A/Virginia/1/2006	A/Solomon Islands/3/2006	0
A/Virginia/1/2006	A/Texas/25/2006	0
A/Brisbane/59/2007	A/Beijing/262/1995	1
A/Brisbane/59/2007	A/Florida/13/2007	0
A/Brisbane/59/2007	A/Florida/19/2007	0
A/Brisbane/59/2007	A/Fukushima/141/2006	0
A/Brisbane/59/2007	A/Pennsylvania/14/2007	0

Supplementary Table S1. Antigenic relationships between antigenic strains of influenza A(H1N1).

*1 represents antigenic variant pairs; 0 represents antigenic similar pairs.

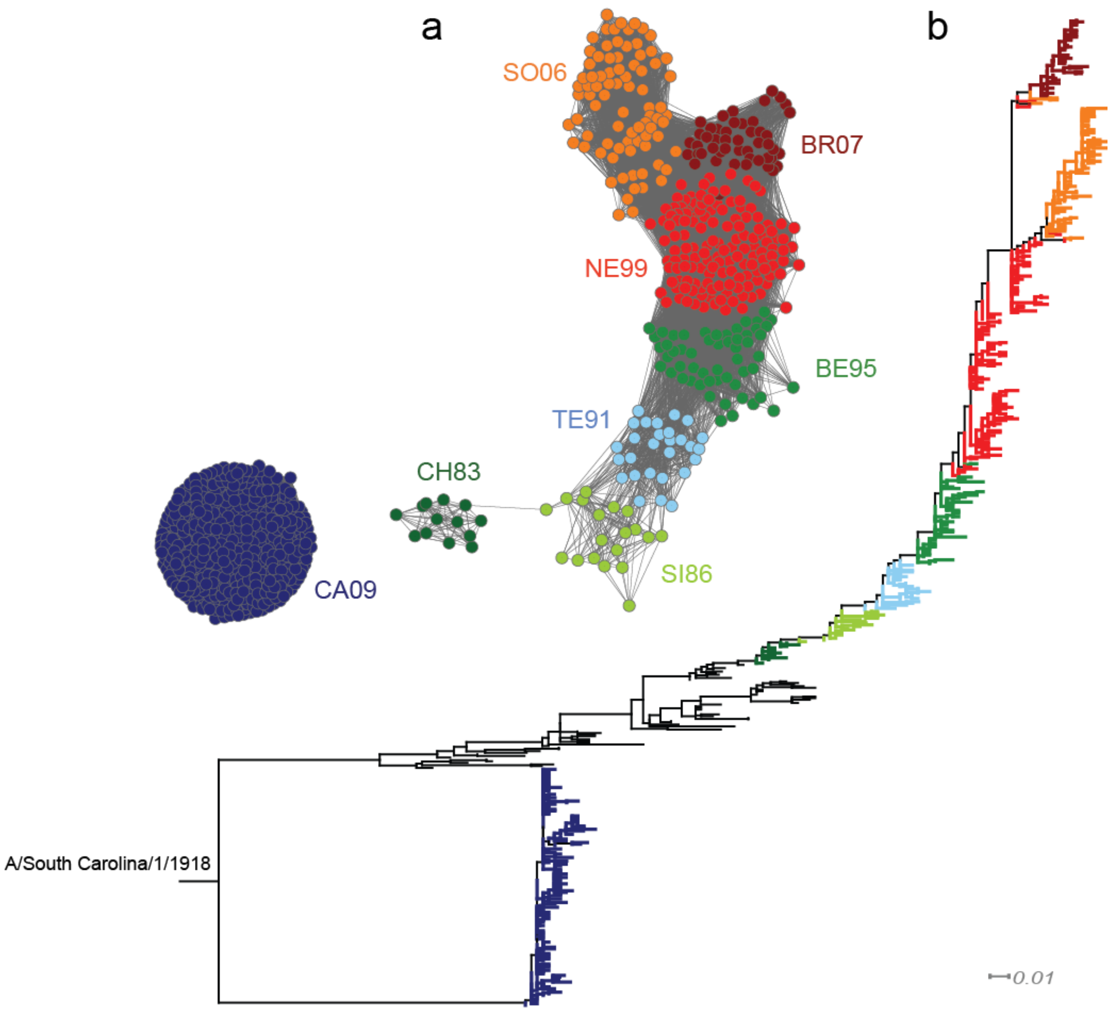

Supplementary Figure S1. Antigenic and genetic evolution of human influenza H1N1 in China from 1977 to 2013. (a) Predicted antigenic correlation network and antigenic clusters of human influenza A (H1N1) viruses in China. (b) Phylogenetic tree of the HA1 protein sequences of viral isolates from China (additional vaccine strains and strains before 1957 from other districts were included).

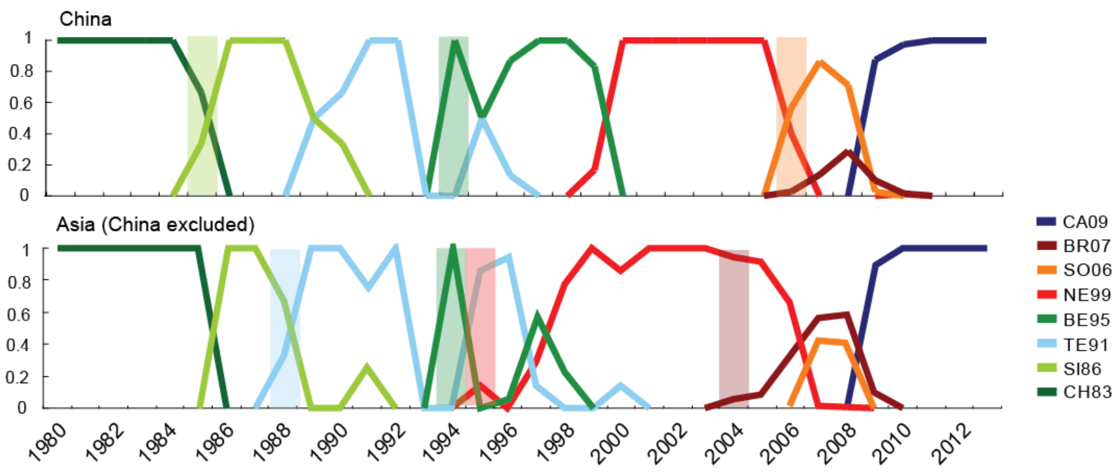

Supplementary Figure S2. Dynamic changes in the percentage of antigenic clusters in China and other areas of Asia.

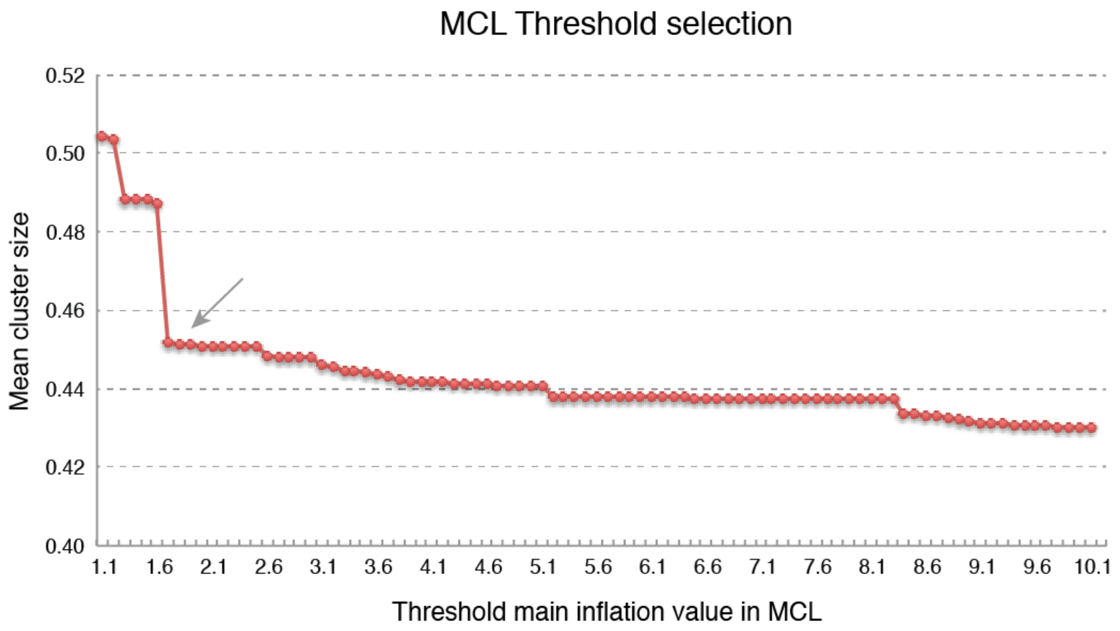

Supplementary Figure S3. Cluster size curve for ACnet clustering of viruses, defined in Du et al.'s work.

Mean cluster size was calculated as the probability of two randomly chosen influenza viruses that lie in the same predicted antigenic cluster. The threshold in the beginning of the first plateau of the cluster size curve (indicated by an arrow) was used for identifying predicted antigenic clusters.

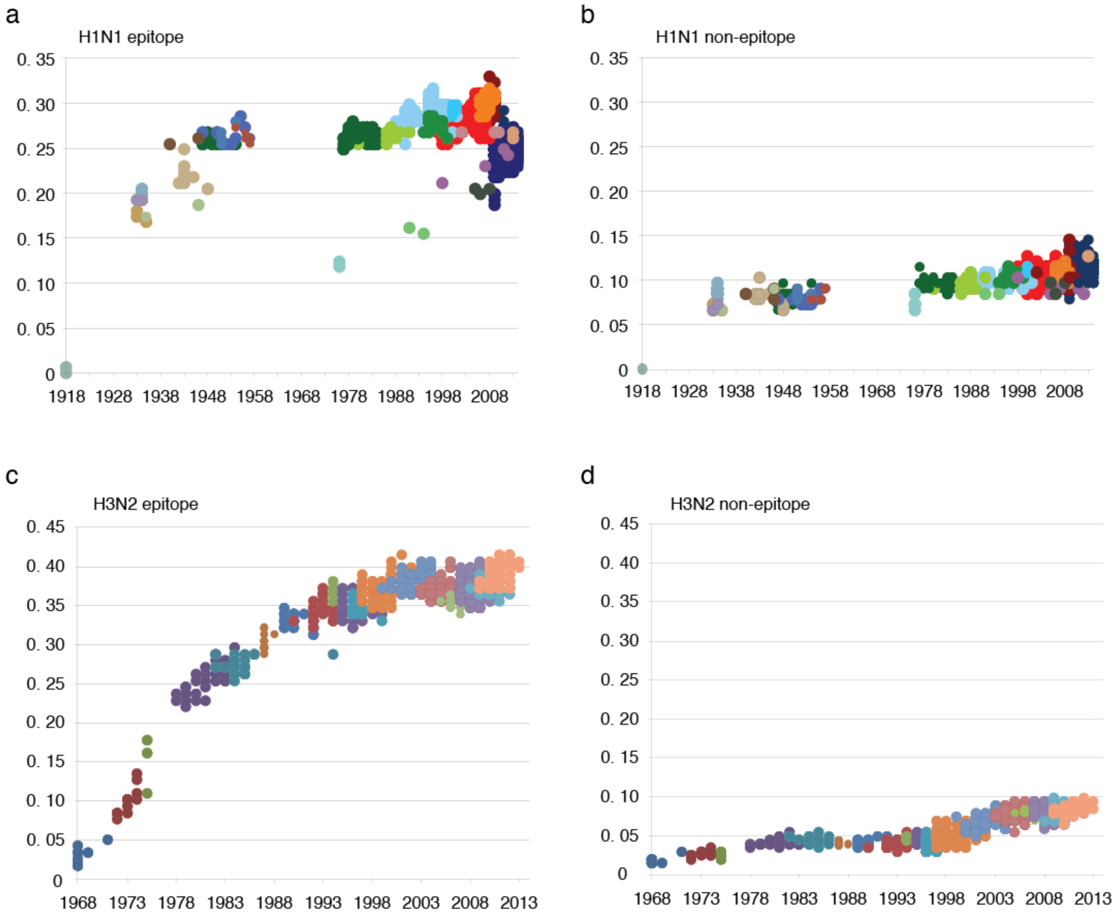

Supplementary Figure S4. (a) Genetic distance on HA1 epitope sites (calculated as amino acid substitutions per site) of human influenza A (H1N1) strains to the A/Brevig Mission/1/1918 (H1N1) virus, each dot represents a virus strain. The strains are colored as antigenic clusters in Fig 2b. (b) Similar as (a), but counted as other sites on HA1 rather than epitope sites. (c) Genetic distance on HA1 epitope sites (calculated as amino acid substitutions per site) of human influenza A (H3N2) strains to the A/Hong Kong/1/1968 (H3N2) virus, each dot represents a virus strain, and strains in the same antigenic cluster were in the same color. (d) Similar as (c), but counted as other sites on HA1 rather than epitope sites.

Supplementary Note S1. PREDAC-H1 shared the same code with PREDAC-H5, which we reported in an earlier paper[6].

Reference

6. Peng, Y., Zou, Y., Li, H., Li, K. & Jiang, T. Inferring the antigenic epitopes for highly pathogenic avian influenza H5N1 viruses. *Vaccine* 1–6 (2014). doi:10.1016/j.vaccine.2013.12.005
